# Metastatic melanoma of the heart: Retrospective cohort study and systematic review of prevalence, clinical characteristics, and outcomes

**DOI:** 10.1002/cam4.5058

**Published:** 2022-07-27

**Authors:** Alexander M. Balinski, Alexi L. Vasbinder, Connor C. Kerndt, Tonimarie C. Catalan, Nathan P. Parry, Rafey A. Rehman, Pennelope Blakely, Raymond Y. Yeow, Monika J. Leja, Christopher D. Lao, Leslie A. Fecher, Salim S. Hayek

**Affiliations:** ^1^ Oakland University William Beaumont School of Medicine Rochester Michigan USA; ^2^ Department of Internal Medicine, Division of Cardiology University of Michigan Ann Arbor Michigan USA; ^3^ Department of Internal Medicine Spectrum Health/Michigan State University College of Human Medicine Grand Rapids Michigan USA; ^4^ Department of Internal Medicine Division of Hematology/Oncology, University of Michigan Ann Arbor Michigan USA

**Keywords:** epidemiology, melanoma, metastasis, prognosis

## Abstract

**Background:**

Cardiac metastasis of melanoma is rare and typically diagnosed post‐mortem. Here we perform a retrospective cohort study and systematic review of patients with metastatic melanoma to characterize prevalence, clinical characteristics, and outcomes of cardiac metastasis.

**Methods:**

We reviewed the electronic medical records of all outpatients with metastatic melanoma who underwent evaluation at the University of Michigan in Ann Arbor from January 2009 to January 2022, identifying patients with a clinical or histopathologic diagnosis of cardiac metastasis. We performed a systematic review of the literature to summarize the clinical characteristics and outcomes of patients with melanoma and cardiac metastasis.

**Results:**

Overall, 23 of 1254 (1.8%) patients with metastatic melanoma were diagnosed with cardiac metastasis. Cardiac metastasis was reported in the right ventricle (65%), left ventricle (35%), and right atrium (35%). A total of 11 (48%) patients experienced at least one cardiovascular complication after the diagnosis of cardiac metastasis, the most common being arrhythmia (30%), heart failure (22%), and pericardial effusion (17%). Immunotherapy was more commonly used in patients with cardiac metastasis (80% vs 65%; *p* = 0.005). Mortality at 2‐years post‐diagnosis was higher for patients with cardiac metastasis compared to those without (59% vs 37%; *p* = 0.034). Progression of malignancy was the underlying cause of death of all patients.

**Conclusions:**

Cardiac metastasis occurs in <2% of patients with metastatic melanoma, can affect all cardiac structures, and is associated with various cardiovascular complications and high mortality.

## Graphical abstract

Cardiac metastasis occurs in 2% of patients with metastatic melanoma, can affect all cardiac structures, and is associated with high mortality. Cardiovascular complications may occur after the diagnosis of cardiac metastasis of melanoma, however with limited impact on mortality, which is predominantly due to progression of malignancy.

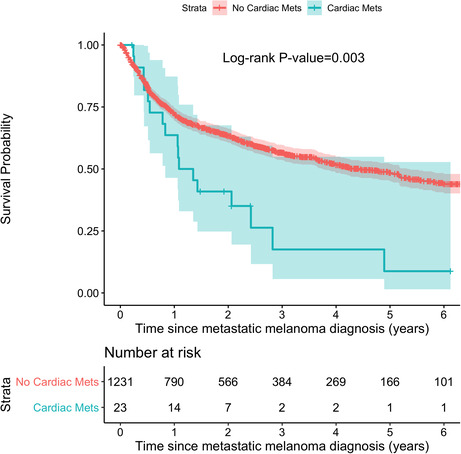

## 1 INTRODUCTION

Melanoma is a highly aggressive malignant tumor. The incidence of melanoma increases linearly after the age of 25 until the age of 50, after which the incidence slows, with a median age of diagnosis of 57 years.^1^ There is a slight predilection of melanoma in men compared with women, with sex‐related differences in survival.^2^, ^3^, ^4^, ^5^, ^6^, ^7^ The advent of targeted therapies and immunotherapy has led to a dramatic improvement in 5‐year survival rates for metastatic melanoma, now greater than 50%.^8^, ^9^ Although melanoma has the potential to metastasize to virtually any organ, common areas of metastasis include the skin, subcutaneous tissue, lymph nodes, lungs, brain, liver, and bone.^10^ Consequently, these tumors may present with a wide variety of symptoms depending on the location of metastasis.

Estimates of the incidence of cardiac metastases vary widely. Most cardiac metastases of melanoma are presumed to be clinically silent and diagnosed post‐mortem, with 28%–64% of metastatic melanoma cases for whom autopsies were performed having heart involvement.^11^, ^12^ Ante‐mortem diagnoses are either incidental or symptomatic and are estimated to occur in <2% of cases.^13^ If identified ante‐mortem, cardiac metastases appear to be associated with a poor prognosis but have demonstrated remission with adequate management.^14^

To date, the characterization of melanoma metastatic to the heart has been limited to case reports and small case series.^12^, ^15^, ^16^ Here we perform a retrospective cohort study of over 1200 patients with metastatic melanoma and a systematic review of the literature to provide a comprehensive analysis of the frequency, clinical presentation, cardiovascular complications, and overall patient outcomes of patients with melanoma and cardiac metastasis.

## 2 MATERIALS AND METHODS

### 2.1 Retrospective cohort study

We conducted a retrospective cohort study of all patients with metastatic melanoma who received outpatient care at the University of Michigan Health System from January 2009 to January 2022. We identified 1254 adult patients (≥18 years) based on referrals to the University of Michigan Melanoma Oncology clinic. Electronic medical records were reviewed to identify patients with melanoma and cardiac metastasis, defined as a diagnosis based on imaging findings (echocardiogram, computed tomography, or magnetic resonance imaging) or histopathology. Data collected included demographics, date of metastatic melanoma diagnosis and treatment, diagnosis and clinical presentation of cardiac metastasis, cardiac imaging and biomarkers, and outcomes. Data were entered into REDCap (Nashville, TN), a secure HIPAA‐compliant web‐based application using a standardized data collection form. This study was approved by the Institutional Review Board at the University of Michigan under a waiver of informed consent.

### 2.2 Systematic review

We performed a systematic review following guidelines set by the Preferred Reporting Systems for Systematic Reviews and Meta‐Analysis (PRISMA).^17^ Studies included in this review were identified after a comprehensive search of the Cochrane Library, EmBase, and PubMed databases from inception until January 2022. The search included keywords related to metastatic melanoma and the heart, such as “metastatic melanoma”, “cardiac metastasis”, “left ventricle”, “right ventricle”, “right atrium”, “left atrium”, “interatrial septum”, and “interventricular septum”. No limitations on dates of publication were placed. Studies were excluded if they were review articles, non‐human, non‐English, or not pertinent to this systematic review. Furthermore, the bibliographies of the relevant studies were searched to identify any additional studies of relevance. Last, the quality of the studies was assessed using the Oxford Center for Evidence Based Medicine (OCEBM) Levels of Evidence categorization.^18^ The titles and abstracts of all the retrieved studies were assessed for inclusion by two authors (AMB and CCK). To be included, the studies must have reported the clinical characteristics of metastatic melanoma to the heart and its management, as well as relevant patient‐level data of confirmed primary melanoma history, symptoms, patient presentation, tumor characteristics, and treatment strategies.

### 2.3 Statistical analysis

We first report clinical characteristics stratified by the presence of cardiac metastasis as means and standard deviation for normally distributed continuous variables and frequencies and proportions for categorical variables. We compared clinical characteristics, treatment modalities, and outcomes between those with and without cardiac metastasis using *t*‐tests or chi‐square tests for continuous and categorical variables, respectively. We created Kaplan–Meier curves to estimate survival associated with the development of cardiac metastasis with follow‐up time beginning at the time of metastatic melanoma diagnosis until death or last known follow‐up date. Differences in survival curves for those who did and did not develop cardiac metastasis were calculated using the log‐rank test.

## 3 RESULTS

### 3.1 Retrospective cohort study

**FIGURE 1:**
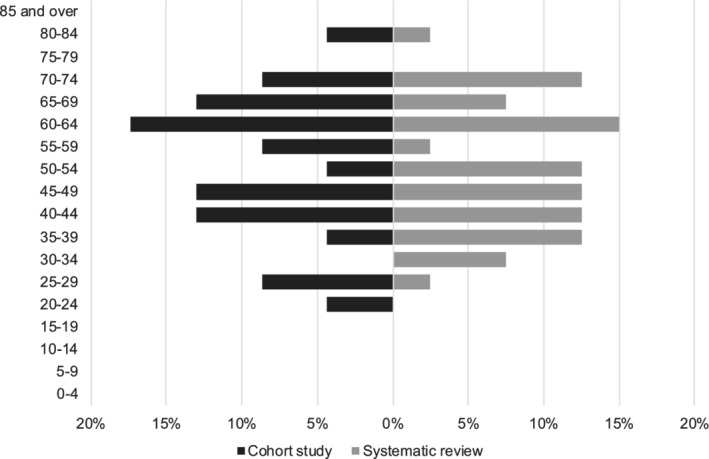
Patient population age distribution of patients with cardiac metastasis of melanoma comparing cohort study (black) vs systematic review (gray).

Overall, we identified 23 (1.8%) cases of melanoma metastasis to the heart out of 1254 patients with metastatic melanoma over a time span of 13 years (2009–2022). Patients with cardiac metastasis consisted of a higher percentage of men (57%), with a mean age at metastatic melanoma diagnosis of 58 ± 16 years (Table 1 and Figure 1). The median (IQR) time from initial melanoma diagnosis to the identification of cardiac metastasis was 2.8 (1.2, 6.1) years. Patients with and without cardiac metastasis had similar demographics and comorbidities at the time of metastatic melanoma diagnosis except for race (Table 1). Compared with patients without known cardiac metastasis, patients with cardiac metastasis were more likely to be black (8.7% vs 0.9%; *p* = 0.009).

**TABLE 1:** Cohort study metastatic melanoma patient comparative analysis

	No cardiac metastasis (*n* = 1231)	Cardiac metastasis (*n* = 23)	*p*‐value
Demographics			
Age at diagnosis, mean (SD)	61 (15)	58 (16)	0.29
Male, *n* (%)	775 (63.0)	13 (56.5)	0.68
Race, *n* (%)			
White	1181 (95.9)	21 (91.3)	0.56
Black/African American	11 (0.9)	2 (8.7)	0.009
Clinical characteristics			
Smoking history, *n* (%)	620 (50.4)	14 (60.9)	0.32
Body mass index, mean (SD)	29 (6)	29 (5)	0.84
Comorbidities, *n* (%)			
Diabetes mellitus	190 (15.4)	2 (8.7)	0.37
Hypertension	515 (41.8)	13 (56.5)	0.16
Heart failure	64 (5.3)	1 (4.3)	0.99
Anemia	72 (5.8)	2 (8.7)	0.90
Hypothyroidism	145 (11.8)	1 (4.3)	0.44
Depression	268 (11.8)	3 (13.0)	0.45
Coagulopathy	81 (6.6)	1 (4.3)	0.99
Primary site, *n* (%)			
Upper extremity	220 (17.8)	4 (17.4)	0.95
Lower extremity	181 (14.7)	2 (8.7)	0.42
Trunk	342 (27.8)	8 (34.8)	0.46
Head/Neck	366 (29.7)	5 (13.0)	0.41
Other	122 (10.0)	4 (17.4)	0.24
Treatments^a^, *n* (%)			
Surgery	67 (5.4)	3 (15.0)	0.10
Targeted therapies	451 (36.7)	7 (35.0)	0.54
Temozolomide	71 (5.8)	2 (10.0)	0.55
Vemurafenib	131 (10.6)	2 (10.0)	0.76
Dabrafenib	305 (24.8)	4 (20.0)	0.42
Trametinib	307 (24.9)	2 (10.0)	0.07
Dabrafenib/Trametinib	286 (23.3)	2 (10.0)	0.10
Immunotherapy	986 (80.1)	13 (65.0)	0.005
Pembrolizumab	467 (37.9)	6 (30.0)	0.25
Ipilimumab	480 (39.0)	9 (45.0)	0.99
Nivolumab	517 (42.0)	4 (20.0)	0.19
Nivolumab/Ipilimumab	246 (20.0)	5 (25.0)	0.83
Radiation	246 (20.0)	5 (25.0)	0.83
Outcomes, *n* (%)			
Mortality at 2‐years	415 (33.7)	13 (56.5)	0.034

^a^ Treatment methods for metastatic melanoma; three patients with cardiac metastasis did not receive treatment (analyses out of 20).

**FIGURE 2:**
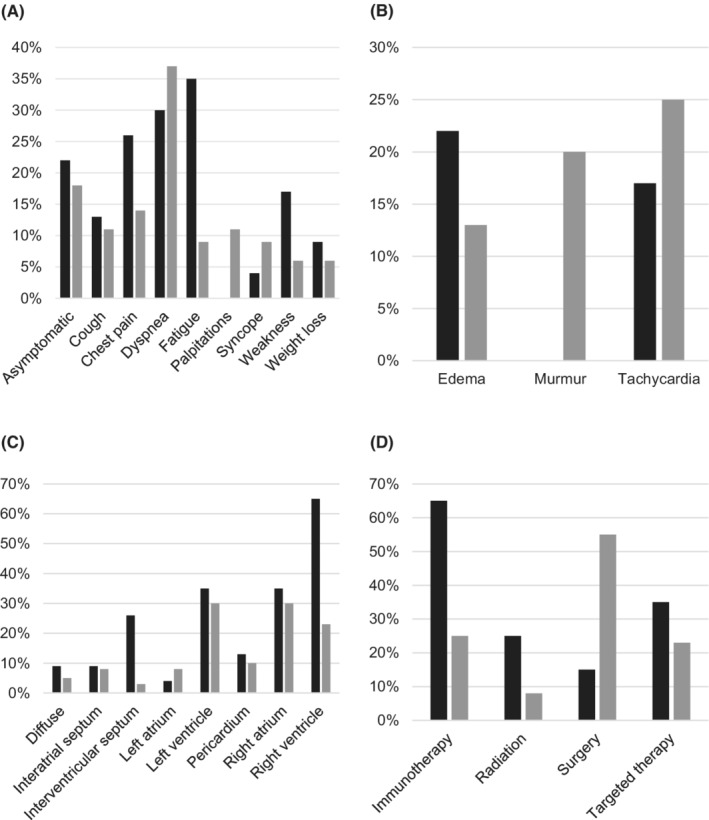
Comparison of the cohort study (black) vs systematic review (gray) patients: (A) symptoms at initial presentation, (B) physical exam findings at initial presentation, (C) cardiac metastasis of melanoma locations, and (D) cardiac metastasis of melanoma treatment strategies.

Close to two‐thirds (61%) of patients had at least one symptom at the time of diagnosis of cardiac metastasis, with the most common presenting symptoms being fatigue (35%) and dyspnea (30%) (Figure 2A). Most patients had no notable cardiac physical exam findings (Figure 2B). Cardiac metastasis commonly presented as a single cardiac mass (65%) in the right ventricle (65%), left ventricle (35%), or right atrium (35%) (Figure 2C). All cases were identified by diagnostic imaging, with a few cases confirmed histologically (13%). Treatment strategies for cardiac metastasis included immunotherapy (65%), targeted therapy (35%), radiation (25%), and surgery (15%) (Table 2, Figure 2D). Patients without cardiac metastasis were more likely to be treated with immunotherapy compared to those with cardiac metastasis (80% vs 65%; *p* = 0.005), specifically nivolumab (42% vs 20%; *p* = 0.018).

**TABLE 2:** Cohort study of detailed patient information

Sex/Age	Primary location	Signs and symptoms	Metastasis locations	Cardiac metastasis treatment	Follow‐up from Cardiac Metastasis diagnosis (months)	Outcomes
F 73	Trunk	Nausea, epigastric pain	LV	Chemotherapy (NOS)	NR	NR
F 72	Nose	CP, epistaxis, weight gain, edema	RA, humerus	Median sternotomy, pericardial window, pembrolizumab, radiation	55	PD, DOD
M 74	Head	SOB, fatigue, nausea, syncope, weakness, diarrhea, hematochezia, back pain, dizziness, tingling, edema	RA, RV, IV septum, stomach	Dacarbazine, ipilimumab	4	PD, DOD
F 31	Lower extremity	Asymptomatic	RV, brain, liver, lung, cervical, thoracic, and lumbar spine, R eye	Radiation	10	PD, DOD
M 46	Back	Headache, R flank pain, tachycardia	RA, IA septum, brain, liver, lung	Dabrafenib, trametinib, ipilimumab, nivolumab, radiation	3	PD, intracranial hemorrhage, DOD
M 72	Upper extremity	Weight loss, L abdominal pain, intermittent night sweats	RV, brain, liver	Temozolomide, vemurafenib, ipilimumab	24	DOD
F 105	Head	Asymptomatic	Pericardium, lung, forehead	NR	10	DOD
M 66	Unknown	Asymptomatic	RV, brain, lung	Dabrafenib, trametinib	12	PD, DOD
M 44	Back	Cough, fatigue, weakness, tachycardia	LV, RV, IV septum, brain, lung, scalp, upper back	Ipilimumab, nivolumab	29	DOD
M 72	L ear	Cough, fatigue, headache	LV, brain, lung, spleen, R axilla	Ipilimumab, nivolumab	NR	NR
M 77	Trunk	Nausea, abdominal pain, constipation, decreased appetite, edema, tachycardia	RV, IV septum, liver, R groin	NR	0	PD, peritoneal carcinomatosis, acute renal failure, DOD
M 54	Trunk	Cough	LA, pericardium, lung, small bowel, thyroid	Pericardial window, ipilimumab	2	PD, DOD
M 72	Upper extremity	Fatigue, nausea, vomiting	RV, liver, lung	Pembrolizumab	16	PD, DOD
F 48	Neck	CP, SOB, fatigue, nausea, migraines, tachycardia	LV, RA, RV, IV septum, brain, liver, L thigh, stomach	Dabrafenib, trametinib, pembrolizumab, ipilimumab	25	DOD
M 59	Upper extremity	CP, SOB, sputum production	LV, RA, RV, IA septum, diffuse myocardium, brain, liver, lung, kidney, spleen	Median sternotomy, pericardial window, ipilimumab, radiation	5	Congestive heart failure, PD, DOD
F 34	Lower extremity	CP, SOB, fatigue, weakness, weight gain, edema	LV, RV, IV septum, brain, lower extremity, upper extremity, neck, kidney	Vemurafenib, dabrafenib, trametinib, melphalan, encorafenib, binimetinib, pembrolizumab, INF alfa‐2b, talimogene laherparepvec	10	PD, DOD
F 54	Back	Altered mental status	Diffuse myocardium, brain, liver, kidney, spleen, spinal cord, eye, humerus, pelvis	NR	1	PD, multifocal cerebral infarction, DOD
F 76	R axilla	Asymptomatic	LV, hip	Pembrolizumab	NR	Remission
M 49	Back	Tinnitus, edema	Pericardium, RA, RV, brain, liver, thoracic spine, kidney, spleen	Temozolomide	2	PD, DOD
F 72	Vulva	Asymptomatic	RV, lymph node, vagina	Radiation	NR	NR
M 71	Trunk	CP, SOB, fatigue, weakness, weight loss, diarrhea	RA, RV, lung, stomach, pancreas, calf	NR	1	PD, DOD
M 48	Upper extremity	CP, SOB, fatigue	LV, RV, liver, pancreas, adrenal glands, spleen	Ipilimumab, nivolumab	NR	NR
F 75	Cervix	SOB	RA, RV, IV septum, lung, uterus, buttocks	NR	2	DOD

Abbreviations: CP, chest pain; DOD, died of disease; F, female; IA, interatrial; IV, interventricular; L, left; LA, left atrium; LV, left ventricle; M, male; NOS, not otherwise specified; NR, not reported; PD, progressive disease; R, right; RA, right atrium; RV, right ventricle; SOB, shortness of breath.

**FIGURE 3:**
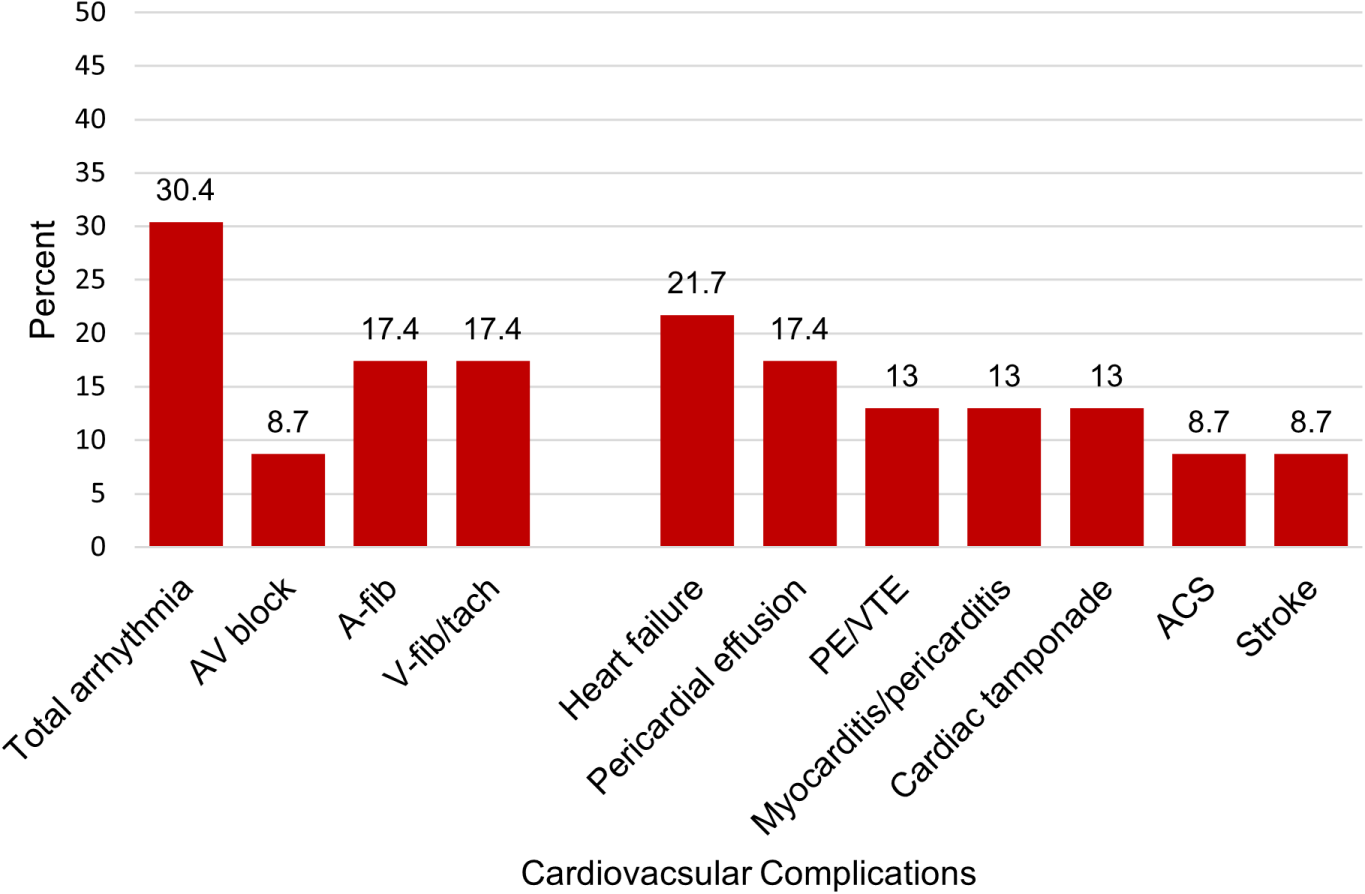
Cumulative incidence of cardiovascular complications within one year after cardiac metastasis. Abbreviations: ACS, acute coronary syndrome; AV, atrioventricular; A‐fib, atrial fibrillation; PE, pulmonary embolism; V‐fib, ventricular fibrillation; V‐tach, ventricular tachycardia; VTE, venous thromboembolism.

**FIGURE 4:**
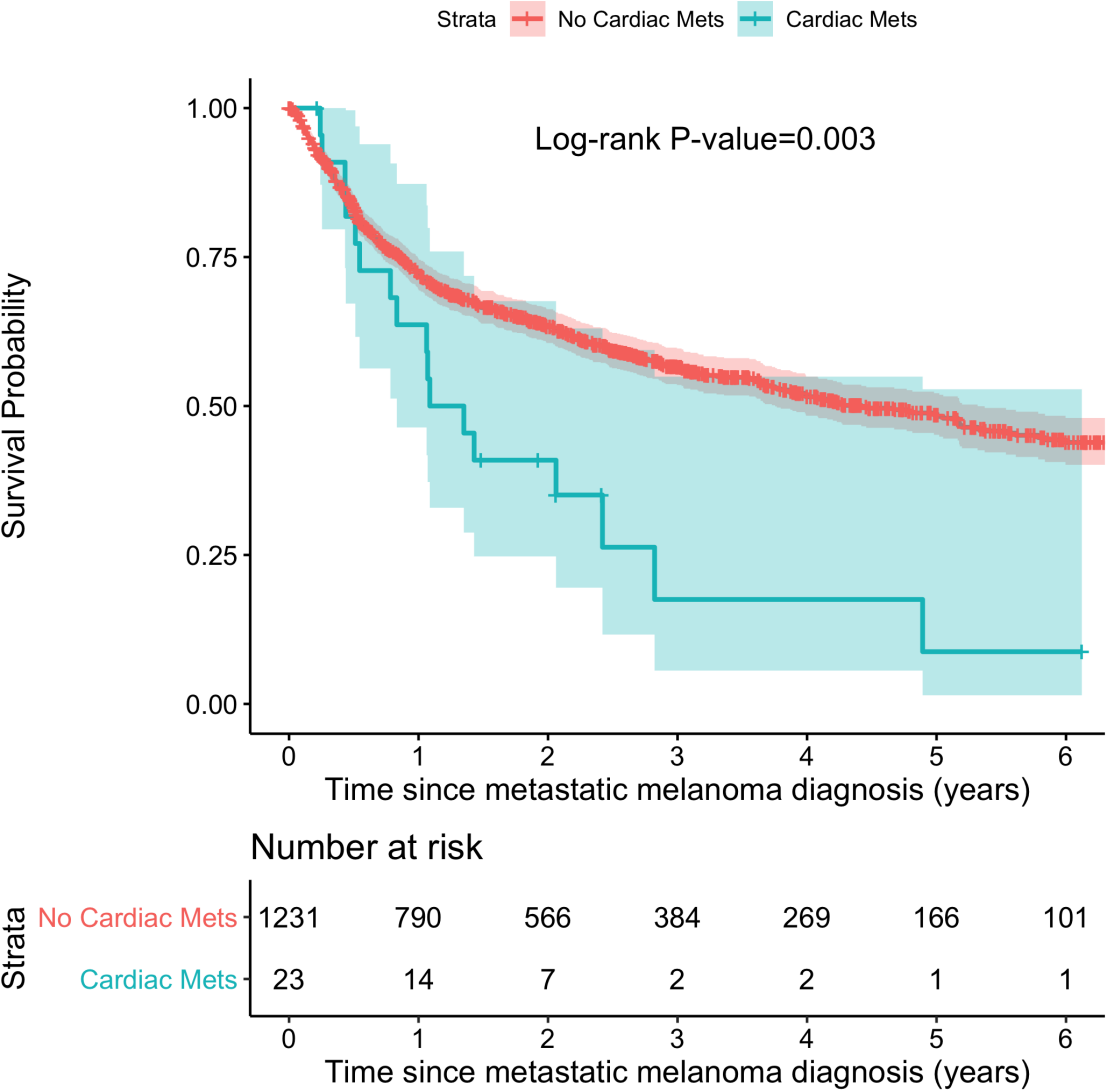
Kaplan–Meier survival curve stratified by cardiac metastasis.

A total of 11 (48%) patients experienced at least one cardiovascular complication within one year after the diagnosis of cardiac metastasis with the most common being arrhythmia (30%), heart failure (22%), and pericardial effusion (17%) (Figure 3). Over a median (IQR) of 1.8 (0.6, 2.4) years, 548 (44%) patients with metastatic melanoma died. Mortality at 1‐year post‐diagnosis of cardiac metastasis was 35%, with 57% of patients having died at two years. The median (IQR) time to death after cardiac metastasis was 0.7 (0.2,1.3) years. At 2‐year post‐metastatic diagnosis, patients with cardiac metastasis had significantly higher mortality compared to patients without cardiac metastasis (59% vs 37%; *p* = 0.034). The median survival time for patients with cardiac metastasis was 1.2 years compared with 4.4 years for patients without cardiac metastasis (Figure 4). Death was attributed to progression of malignancy in all causes, with no deaths attributed to cardiovascular causes.

### 3.2 Systematic review

Our systematic literature review identified 1012 articles with 36 studies meeting inclusion criteria (Figure S1).^19^, ^20^, ^21^, ^22^, ^23^, ^24^, ^25^, ^26^, ^27^, ^28^, ^29^, ^30^, ^31^, ^32^, ^33^, ^34^, ^35^, ^36^, ^37^, ^38^, ^39^, ^40^, ^41^, ^42^, ^43^, ^44^, ^45^, ^46^, ^47^, ^48^, ^49^, ^50^ All studies received an evidence level of 4 based on the OCEBM Levels of Evidence categorization with a total of 40 individual cases of cardiac metastasis of melanoma identified from these studies.

Patients consisted of 50% men, with a mean age of presentation of 52 ± 14 years and a mean time from primary melanoma diagnosis to diagnosis of cardiac metastasis of 8 ± 8 years. Patients most commonly presented with dyspnea (40%) and non‐specific physical exam findings (Figure 2A,B). Cardiac metastasis was most often identified as a single mass (83%) located in the left ventricle (30%), right atrium (30%), or right ventricle (23%) (Figure 2C). Most cases were histologically confirmed (80%) and treatment strategies most often involved surgical intervention (55%) or immunotherapy (25%) (Figure 2D). Twenty‐eight (70%) cases reported outcomes. When reported, follow‐up times ranged from 0 to 5 years, with an average of 0.8 ± 1.3 years. A total of 26 (65%) cases described at least one cardiovascular complication within one year after the diagnosis of cardiac metastasis, with the most common being right ventricular obstruction (40%), arrhythmia (35%), and pericardial effusion (20%). Of the 11 (39%) patients who were reported deceased, the majority (91%) perished within one year of cardiac metastasis diagnosis with 2 (18%) deaths due to cardiovascular complications. Detailed descriptions of patient findings from the systematic review are further depicted in Table S1.

### 3.3 Retrospective cohort study and systematic review comparison

Patient demographics were similar between our cohort study and systematic review. Presenting symptoms and physical exam findings were generally non‐specific. Patients commonly presented with one cardiac tumor but our cohort study showed cardiac metastasis presented most often in the right ventricle (65%), whereas our systematic review showed cardiac metastasis presented most often in the left ventricle (30%) and right atrium (30%). Treatment with immunotherapy was more common in our cohort study compared with our systematic review (65% vs 25%), whereas surgical intervention was used more often in our systematic review (55% vs 15%). Interestingly, mortality at 2‐years was higher in our retrospective cohort study (59%) compared with our systematic review (39%).

## 4 DISCUSSION

In this retrospective cohort study of 1254 patients with metastatic melanoma over a 13‐year period, cardiac metastasis occurred in <2% of patients and was associated with significantly worse mortality compared to patients with non‐cardiac metastasis. Patients presented with a wide variety of symptoms and non‐specific physical exam findings. Metastasis did not have a predilection for a specific cardiac structure, and a variety of cardiovascular complications occurred including arrhythmias, heart failure, and pericardial effusions. Treatment of cardiac metastasis of melanoma commonly involved immunotherapy. Our systematic review of 1012 articles identified 40 additional cases which mostly supported the findings of our cohort study. The findings of our study highlight that cardiac metastasis from melanoma is an aggressive manifestation of this malignancy that can be difficult to identify clinically and is associated with worse outcomes.

Cardiac metastasis of melanoma can manifest as multiple small tumors, large masses, or infiltrative disease.^12^, ^51^, ^52^, ^53^ While other malignancies tend to involve the pericardium and epicardium, melanoma appears to have a particular propensity for endocardial involvement for reasons that are unknown.^16^ While metastasis can involve any chamber of the heart, past studies have demonstrated that the right atrium is the most common location for cardiac involvement from metastatic melanoma.^12^, ^13^, ^54^ Intracavitary metastases to the right atrium have been suggested to occur as a result of diffusion along the inferior vena cava, subsequently producing the intracavitary lesion.^11^ Less frequently reported areas of cardiac metastases are the right ventricle and the left atrium.^13^ Metastases to the left atrium are thought to arise in patients with lung cancer whose tumors embolize through the pulmonary circulation to the left atrium.^11^ We found the most common locations of cardiac metastasis from melanoma included the left ventricle, right ventricle, and right atrium. To our knowledge, the involvement of the left ventricle has not been previously reported and may be related to the increased myocardial mass and vascularization of the left ventricle, which cardiac metastases of other malignancies have demonstrated a preference for.^55^

Cardiac involvement from metastatic melanoma typically occurs without specific clinical manifestations and is usually diagnosed post‐mortem as a result.^12^ Cases that are discovered ante‐mortem are either incidental or symptomatic and cause non‐specific symptoms such as fatigue, chest pain, exertional dyspnea, or leg edema, prompting a detailed cardiac evaluation.^13^ Clinical findings are often a result of cardiac metastasis localization with arrhythmia due to conduction system or endomyocardial infiltration, heart failure due to an obstructive mass, and pericardial effusion.^12^, ^56^, ^57^ In this study, the most common presenting symptoms were fatigue, dyspnea, and chest pain. Physical exam findings such as distant heart sounds, new murmurs, or a pericardial friction rub^11^ may also be associated with cardiac involvement of metastatic melanoma but patients in this study did not demonstrate such specific physical exam findings.

Once diagnosed, the prognosis of cardiac metastasis from melanoma is poor. The average time between diagnosis of cardiac metastasis from melanoma and death has been reported to be two years.^13^ Despite the poor prognosis, immunotherapy, targeted therapy, radiotherapy, and/or surgery can be useful to alleviate symptoms and reduce their cardiovascular impact.^52^, ^58^, ^59^, ^60^ While many patients in our retrospective cohort experienced at least one cardiovascular complication after the diagnosis of cardiac metastasis, all reported deaths were due to the progression of malignancy. Accordingly, the higher mortality in patients with cardiac metastasis compared to those without is likely reflective of a higher tumor burden rather than a direct impact on the cardiovascular system.

In our systematic review, surgery was the main strategy utilized for tumor management and many patients experienced remission when surgery was implemented with the addition of other treatment options. Some cases opted for immunotherapy or targeted therapy as singular or combined therapies for patient treatment. Two patients died due to cardiovascular complications in our systematic review and both instances involved procedures (left ventriculotomy, endomyocardial biopsy) that may have further destabilized the integrity of a fragile myocardium, resulting in myocardial rupture. As some of our cases and other case reports predate the most modern and effective immunotherapies and targeted therapies, it is still to be determined how these therapies will impact the outcomes of patients with cardiac metastasis in the future.

### 4.1 Limitations

We sought to provide an estimate of incidence by performing a retrospective cohort study. Given the lack of systematic testing for cardiac metastasis or autopsy data, the incidence we report is likely an underestimate. We found a significantly higher 2‐year mortality in patients with cardiac metastasis. However, the relatively small number of cases precludes performing stratified or multivariable analyses. While it is likely that the higher mortality in patients with cardiac metastasis reflects an increased tumor burden, the lack of systematic assessment of tumor burden precludes us from making definite conclusions. Similarly, findings from the cohort and systematic review suggest an endocardial rather than pericardial predilection of metastatic melanoma, however, these conclusions are not definite in the absence of systematic testing such as cardiac MRI or biopsy. Last, findings related to differences in race are exploratory at best and warrant validation.

## 5 CONCLUSION

This is the largest cohort study investigating the prevalence, clinical presentation, treatment, and outcomes of metastatic melanoma of the heart and provides a contemporary characterization of the disease. Cardiac metastasis occurs in <2% of patients with metastatic melanoma but is associated with high mortality. Patients may present with a wide variety of symptoms, and clinicians should maintain a high index of suspicion for cardiac metastasis when patients present with fatigue, dyspnea, and a history of melanoma. Cardiac metastasis of melanoma may involve any chamber of the heart and a predilection for a specific cardiac structure is unclear. Treatment strategies typically involve immunotherapy, targeted therapy, radiotherapy, or surgical intervention when indicated. Cardiovascular complications may occur after the diagnosis of cardiac metastasis of melanoma, however, with limited impact on mortality, which is predominantly due to progression of malignancy.

## AUTHORS’ CONTRIBUTION

**Alexander M. Balinski:** Conceptualization, data curation, formal analysis, investigation, methodology, writing—original draft, and writing—review and editing; **Alexi L. Vasbinder:** Data curation, formal analysis, methodology, writing—original draft, and writing—review and editing; **Connor C. Kerndt:** Data curation, writing—original draft, and writing—review and editing; **Tonimarie C. Catalan:** Data curation; **Nathan P. Parry:** Formal analysis; **Rafey A. Rehman:** Data curation; **Pennelope Blakely:** Data curation; **Raymond Y. Yeow:** Writing—review and editing; **Monika J. Leja:** Writing—review and editing; **Christopher D. Lao:** Writing—review and editing; **Leslie A. Fecher:** Writing—review and editing; **Salim S. Hayek:** Conceptualization, formal analysis, investigation, methodology, project administration, supervision, writing—original draft, and writing—review and editing.

## FUNDING INFORMATION

ALV is supported by an NHLBI‐funded postdoctoral fellowship (T32HL007853). SSH is funded by the NHLBI (1R01HL153384‐01) and the NIDDK (1R01DK12801201A1).

## CONFLICT OF INTEREST

None.

## Supporting information


Figure S1

Table S1

## Data Availability

The data that support the findings of this study are available on request from the corresponding author. The data are not publicly available due to privacy or ethical restrictions.
