# Supplementary material for: Metastatic melanoma of the heart: Retrospective cohort study and systematic review of prevalence, clinical characteristics, and outcomes
Source: Cancer Med. 2022 Jul 27;12(3):2356–67. doi: 10.1002/cam4.5058 (PMC9939187; doi:10.1002/cam4.5058)
Supplement: Supplementary file 1 — Figure S1 [file CAM4-12-2356-s002.pdf]

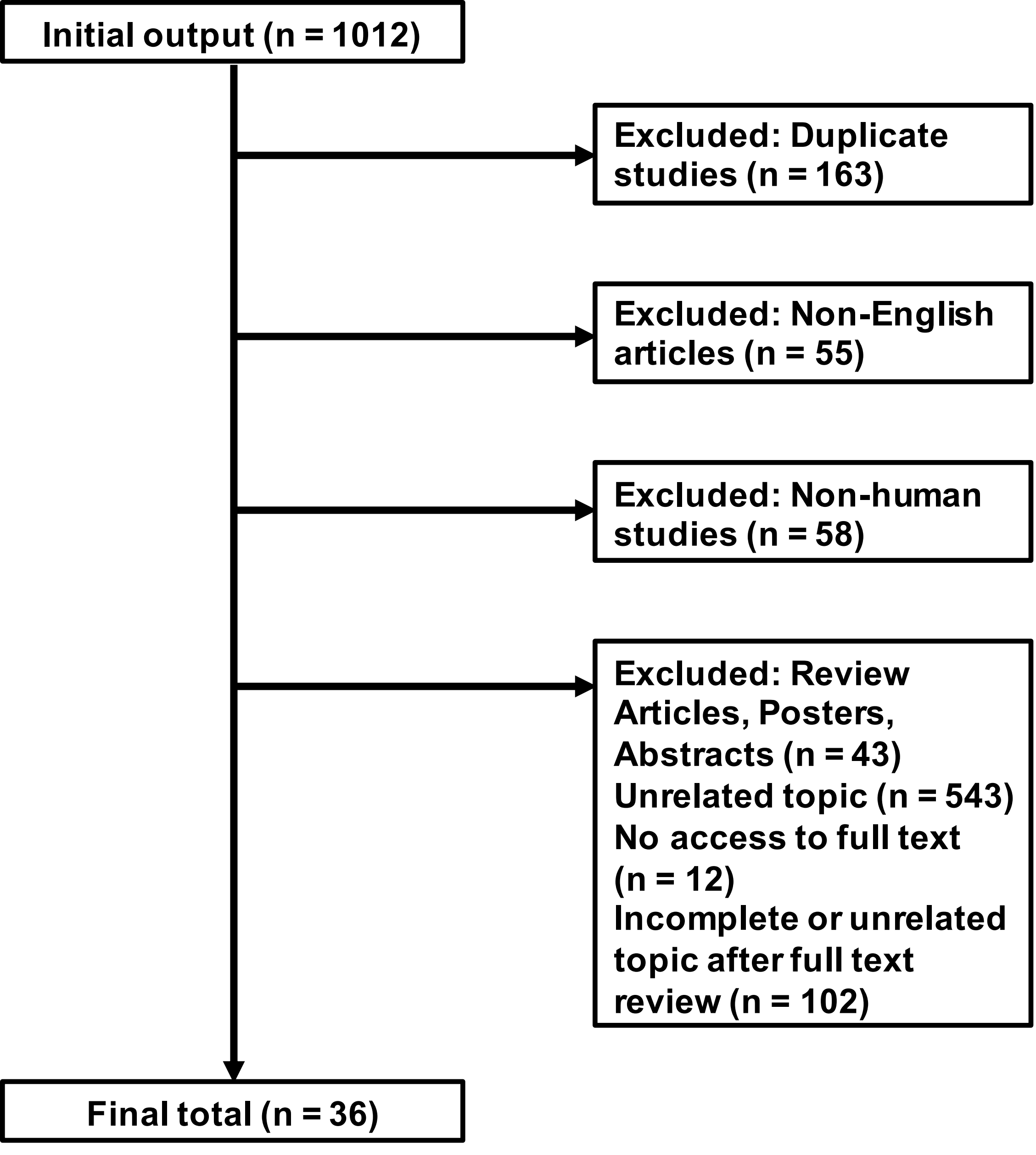
Initial output (n = 1012)
Excluded: Duplicate studies (n = 163)
Excluded: Non-English articles (n = 55)
Excluded: Non-human studies (n = 58)
Excluded: Review Articles, Posters, Abstracts (n = 43)
Unrelated topic (n = 543)
No access to full text (n = 12)
Incomplete or unrelated topic after full text review (n = 102)
Final total (n = 36)
