# Supplementary material for: Metastatic melanoma of the heart: Retrospective cohort study and systematic review of prevalence, clinical characteristics, and outcomes
Source: Cancer Med. 2022 Jul 27;12(3):2356–67. doi: 10.1002/cam4.5058 (PMC9939187; doi:10.1002/cam4.5058)
Supplement: Supplementary file 2 — Table S1 [file CAM4-12-2356-s001.docx]

| **Supplemental Table 1. Systematic review detailed patient information** | | | | | | | | |
| --- | --- | --- | --- | --- | --- | --- | --- | --- |
| **Authors** | **Age/Sex** | **Primary Location** | **Signs & Symptoms** | **Metastases Locations** | **Histopathologic Findings** | **Treatment** | **Follow-up (months)** | **Outcomes** |
| Gibbs^20^ | F 46 | L neck | CP, SOB, subcutaneous mass, tachycardia | RA prolapsing into RV | Metastatic melanoma | Surgery (NOS) | 6 | Alive, slow progression of soft tissue metastases, asymptomatic |
| Malouf^21^ | M 73 | L axilla | Asymptomatic | RA | Malignant cells enlarged, eccentric nuclei with large macronucleoli and occasional intranuclear inclusions of cytoplasm; in rare cells, golden brown granular cytoplasm, positive for S-100 and HMB-45 | IL-2, levamisole | NR | NR |
| Emmot^24^ | M 60 | Nose | SOB, edema, grade III/VI harsh systolic ejection murmur | RV anterior free wall | Confirmed melanoma | Surgery (NOS) | NR | Excellent post-operative relief, remission |
| Houmsse^25^ | M 42 | Leg | SOB, grade II/VI systolic ejection murmur | LV apex, lung | Positive for S-100 and Melanin | Median sternotomy, chemotherapy (NOS), IL-2 | 2 | Asymptomatic, remission |
| Rosario^29^ | F 84 | L choroid | Syncope, grade III/VI crescendo-decrescendo systolic murmur | LV | Spindle and epithelioid cells, some of which were arranged in a fascicular pattern, consistent with malignant melanoma | Left ventriculotomy | 0 | Mediastinal hemorrhage, cardiovascular collapse, deceased |
| Kolandjian^30^ | M 38 | R uvea | CP, SOB, nausea, bradycardia, distant heart sounds, S4 | Posterior inferior LV, liver | NR | BOLD | NR | Remission |
| Jakate^31^ | M 68 | L ocular | SOB, systolic ejection murmur | LV, RA, liver | Pleomorphic nuclei with abundant pigment, spindled and epithelioid | Debulking | NR | NR |
| Ruiz^32^ | F 74 | L choroid | Dizziness | LV | Pigmented cells invading the myocardium | Excisional biopsy | 0 | Ruptured myocardium, deceased |
| Faustino^33^ | F 47 | L choroid | Pleuritic CP | Diffuse pericardium, myocardium | Positive for HMB-45 and S-100 protein | Dacarbazine | 36 | Cardiac remission, brain metastasis, DOD |
| Catapano-Minotti^34^ | F 38 | Thigh | Exertional dyspnea, fatigue, syncope, tachycardia, grade II/VI systolic murmur (tricuspid/pulmonic) | RV outflow tract (infundibulum) | Melanoma cells | Surgical excision | NR | NR |
| Bertella^35^ | F 38 | R hemithorax | Cough, CP, SOB, orthopnea, edema, tachycardia | LV | BRAF (+) | Pericardial window, trametinib, dabrafenib | NR | Remission |
| Park^36^ | F 53 | R shoulder | SOB, tachycardia, palpitations, diminished heart sounds | LV | Reactive mesothelial cells and chronic inflammation | Pericardial window, chemotherapy (NOS) | 2 | Pericardial fluid return, remission |
| Krüger^37^ | M 41 | L foot | Asymptomatic | RA, SVC confluence | Intracardiac metastasis of a malignant melanoma | Median sternotomy | NR | NR |
| Tas^38^ | F 44 | Occiput | Asymptomatic | Fossa ovalis, IA septum, liver, lung, bone, soft tissue | NR | Fotemustine, temozolomide, IFN alfa-2b | NR | NR |
| Mousseaux^39^ | M 46 | R elbow | Cardiac tamponade | LA, LV, liver, lung | NR | Carboplatin, IFN alfa-2b | NR | NR |
|  | M 67 | L thigh | Palpitations | LV | Achromic malignant melanoma | Surgical ablation | 60 | Remission |
|  | M 50 | Neck | SVC syndrome | RA into SVC | NR | Surgical ablation | 12 | Remission |
|  | F 63 | Back | Asymptomatic | LA | NR | Fomustine | NR | NR |
| Pedrotti^40^ | M 47 | Back | SOB, tachycardia | RA, RA septum | Epithelioid-type melanoma, BRAF (+) | Vemurafenib | NR | NR |
| Kontozis^41^ | F 51 | L breast | Fatigue, headache, malaise, tachycardia | RA, breast | Epithelioid cell proliferation with a generally nodular pattern of growth, areas of ischemic necrosis, moderate cytologic pleomorphism and marked melanin-type pigmentation | Median sternotomy | 5 | No recurrence, remission |
| Kumar^42^ | F 74 | R plantar toe | Progressive fatigue, malaise, weight loss | RA, ascending colon | Metastatic melanoma, positive for S-100, HMB-45, and Melan-A | Surgery (NOS) | NR | NR |
| Roubille^43^ | M 61 | Thorax | Cough, SOB, tachycardia, systolo-diastolic murmur | RV | Obviously malignant tumor with focal dark brown intracytoplasmic granules, positive for S-100 protein, HMB-45, and Melan-A antibodies | Dacarbazine | 3 | Stabilized lesion, DOD |
| Mindell^44^ | F 65 | Back | Exertional dyspnea, fatigue, edema | RV, RV septum, brain | Poorly differentiated malignant neoplasm. Tumor cells in solid sheets and masses with scant supporting stroma. Hyperchromatic pleomorphic nuclei with occasional mitotic figures. S-100 (+) | Surgery (NOS) | 5 | Congestive heart failure and tumor regrowth, DOD |
| Magnuson^45^ | M 63 | Nasal cavity | Cough, hemoptysis | LA into pulmonary vein, brain, liver | Consistent with melanoma | Radiation | 9 | Liver metastasis, DOD |
| Judge^46^ | F 36 | Abdomen | Asymptomatic | RV, liver | NR | RV metastasectomy, temozolomide, IL-2 | NR | Liver complications and cardiac tumor recurrence, DOD |
|  | M 49 | Thigh | SOB | IA septum | NR | Debulking | NR | Cardioembolic stroke, palliative care, DOD |
| Cicin^47^ | F 33 | L shoulder | Throat discomfort, bleeding | RA, tonsil, gallbladder | Consistent with melanoma | Surgery (NOS), temozolomide | 9 | No metastasis, remission |
| Khan^48^ | M 27 | L thumb | Testicular swelling, blue body lesions, weight loss, night sweats, rigors, chills | Diffuse LA, RA, LV, RV, testicle, iris, skin | Positive for Melan-A, S-100, and HMB-45 | Dacarbazine, cisplatin, IL-2 | NR | Reduction of metastasis, remission |
| Safa^49^ | F 61 | L great toe | Asymptomatic | LV | Tumor cells, melanocytes, SOX-10 (+) | Pembrolizumab | 15 | Resolution of metastasis, remission |
| Merer^50^ | M 34 | R neck | New subcutaneous nodule, I/VI early systolic tumor plop, III/IV diastolic rumble | RV, subcutaneous chest wall, brain | Enlarged pleomorphic nuclei, dark granular cytoplasm, mitotic figures, melanin | Median sternotomy non-bypass | NR | NR |
| Ellis^51^ | M 63 | Back | Lethargy, fatigue, night sweats | LV | Melanoma | Radiation, chemotherapy (NOS) | 2.5 | Melanosis, DOD |
| Beliaev^52^ | M 71 | R shin | SOB | IA septum, small bowel, peritoneum | Malignant epithelioid cells, small spindle cells | Complete excision with AICD placed, pembrolizumab | NR | Remission |
| Babar^53^ | M 57 | R shoulder | CP, SOB, palpitations, tachycardia | RV | Malignant pleomorphic spindle cell neoplasm infiltrating cardiac muscle and pericardium, S-100 (+), SOX-10 (+) | Complete resection with RV free wall and RV outflow tract reconstruction with bovine pericardial patch, ipilimumab/nivolumab | 3 | Remission |
| Haiduk^54^ | F 39 | Uvea | Asymptomatic | RV | Malignant melanoma | Resection, cryoablation, nivolumab | 9 | Remission |
| Poggi^55^ | F 41 | L choroid | Asymptomatic | LV, brain, liver, lung | NR | Temozolomide | NR | NR |
| Friedel^56^ | F 40 | R shoulder | Recurrent arrhythmia, edema, tachycardia, systolic murmur apex | IV septum | NR | Vemurafenib | NR | Brief remission |
| Fontana^57^ | M 72 | Back | SOB, atypical CP | RV | NR | Vemurafenib | NR | NR |
| Durham^58^ | F 50 | Chest | Lower extremity numbness, visual auras, handwriting changes, SOB, fever, fatigue, weakness, weight loss, S3 | RA, conus medullaris, intraparenchymal lesions | NR | Vemurafenib | 6 | Hospice, DOD |
| Burn^59^ | F 33 | L forearm | SOB | RA, L supraclavicular node, axillary node, L adrenal gland | NR | Chemotherapy (NOS) | NR | Mass enlarged to 4 cm |
| Steger^60^ | M 53 | L nasal cavity | L sided epistaxis, nasal obstruction | Pericardium, liver, lung, thyroid, stomach, pancreas, colon, peritoneal cavity, urinary bladder, bones, jugular lymph node, omental lymph node, mesenteric lymph node, chest wall, mandible | NR | Dacarbazine | 1 | Worsening abdominal pain, DOD |
| Abbreviations: BOLD, bleomycin, vincristine, lomustine, dacarbazine; CP, chest pain; DOD, died of disease; F, female; HMB-45, human melanoma black 45; IA, interatrial; IFN, interferon; IL-2, interleukin-2; IV, interventricular; L, left; LA, left atrium; LV, left ventricle; M, male; NOS, not otherwise specified; NR, not reported; R, right; RA, right atrium; RV, right ventricle; SOB, shortness of breath; SOX-10, SRY-related HMG-box 10; SVC, superior vena cava. | | | | | | | | |
